# A review of the genus *Lankaphthona* Medvedev, 2001, with comments on the modified phallobase and the unique abdominal appendage of *L.binotata* (Baly) (Coleoptera, Chrysomelidae, Galerucinae, Alticini)

**DOI:** 10.3897/zookeys.857.34465

**Published:** 2019-06-25

**Authors:** Yongying Ruan, Alexander S. Konstantinov, Kaniyarikkal D. Prathapan, Mengna Zhang, Xingke Yang

**Affiliations:** 1 College of Agriculture, South China Agricultural University, Guangzhou, Guangdong 510642, China South China Agricultural University Guangzhou China; 2 School of Applied Chemistry and Biological Technology, Postdoctoral Innovation Practice Base, Shenzhen Polytechnic, Shenzhen, Guangdong 518055, China Postdoctoral Innovation Practice Base Shenzhen China; 3 Systematic Entomology Laboratory, USDA, ARS, Washington DC, USA Systematic Entomology Laboratory, USDA Washington United States of America; 4 Department of Entomology, Kerala Agricultural University, Vellayani P.O., Trivandrum -695 522, Kerala, India Kerala Agricultural University Kerala India; 5 Key Laboratory of Zoological Systematics and Evolution, Institute of Zoology, Chinese Academy of Sciences, Beijing 100101, China Institute of Zoology, Chinese Academy of Sciences Beijing China

**Keywords:** abdominal process, flea beetles, new combinations, new synonyms, sexual dimorphism, spiculum, tegmen

## Abstract

The flea beetle genus *Lankaphthona* Medvedev, 2001 is redescribed and a new species *L.yunnantarsella***sp. nov.** Ruan, Konstantinov & Prathapan is described. *Longitarsella* Medvedev, 2009, **syn. nov.** and *Philotarsa* Medvedev, 2009, **syn. nov.** are newly synonymized with *Lankaphthona*. *Philotarsalaosica* Medvedev, 2009, **syn. nov.** is synonymized with *Lankaphthonaphuketensis* (Gruev, 1989). The following new combinations are proposed: *Lankaphthonabinotata* (Baly, 1876), **comb. nov.**; *Lankaphthonacostata* (Medvedev, 2016), **comb. nov**.; *Lankaphthonacyanipennis* (Medvedev, 2017), **comb. nov.**; *Lankaphthonanigronotata* (Jacoby, 1896), **comb. nov.**; *Lankaphthonanotatipennis* (Medvedev, 2009), **comb. nov.**; and *Lankaphthonaphuketensis* (Gruev, 1989), **comb. nov., status restored**. A highly specialized spoon-shaped ‘appendage’ is discovered on the first abdominal ventrite of males of *Lankaphthonabinotata*. Aedeagus of the same species has aberrant sheath-shaped phallobase encircling the median lobe. Morphology and possible function of these structures are discussed. Menispermaceae are newly reported as the host plants of the genus.

## Introduction

*Lankaphthona*, established by Medvedev (2001) for three new species from Sri Lanka, belongs to a hypothetically monophyletic group of Oriental genera, characterized by a pair of subparallel longitudinal ridges on the first abdominal ventrite. In addition to *Lankaphthona*, this generic group includes *Philogeus* Jacoby, 1887, *Tegyrius* Jacoby, 1887, *Lanka* Maulik, 1926, *Bikasha* Maulik, 1931, *Parategyrius* Kimoto & Gressitt, 1966, *Neorthana* Medvedev, 1996, *Lankanella* Kimoto, 2000, *Longitarsella* Medvedev, 2009, *Philotarsa* Medvedev, 2009 and *Lesagealtica* Döberl, 2009. These genera also share the following character states: small to moderate size, body oblong and convex in lateral view, procoxal cavities open, metatibia with distal end outwardly curved and dorsal surface almost flat.

*Longitarsella* was proposed by Medvedev (2009) as a subgenus of *Trachyaphthona* Heikertinger (Konstantinov and Prathapan (2008) synonymized *Trachyaphthona* with *Trachytetra* Sharp) for two species with elongate first metatarsomere: *L.notatipennis* Medvedev, 2009 and *Thyamisbinotata* Baly, 1876, with the latter designated as the type species of the subgenus (Medvedev 2009). However, upon specimen examination it became clear that what Medvedev (2009) identified as *L.binotata* (Baly) is a case of misidentification and, in fact, exactly the same species that Medvedev described (in the same work 60 pages prior) as Philopona (Philotarsa) laosica Medvedev, 2009, which he designated as the type species of *Philotarsa* (as a subgenus of *Philopona*).

*Longitarsella* and *Philotarsa* share the same type species and therefore are objective synonyms. Additionally, *Philotarsa* is dramatically different from *Philopona* Weise and *Longitarsella* is very different from *Trachytetra* Sharp, so that discussion on *Philopona* and *Trachytetra* is a separate topic in flea beetle taxonomy and nomenclature.

Further studies on *Lankaphthonamicheli* Medvedev, 2001 (the type species of *Lankaphthona*), *Longitarsellanotatipennis* Medvedev, 2009 and *Philotarsalaosica* confirmed that all three species are clearly congeneric. Since *Lankaphthona* is the oldest available name, *Philotarsa* and *Longitarsella* are here synonymized with it. In addition to the six species treated in this study, Medvedev described two more species of *Longitarsella*: *L.costata* Medvedev, 2016 from Malaysia and *L.cyanipennis* Medvedev, 2017 from Indonesia. Since *Longitarsella* is no longer valid, these species are here transferred to *Lankaphthona*. So far, eight species are known in the genus *Lankaphthona*. However, the types of the latter two species are not available for this study. Since they cannot be properly treated and included in a species key based on the information provided in the descriptions (Medvedev 2016, 2017), they are treated as species *incertae sedis* here.

**Figure 1.**
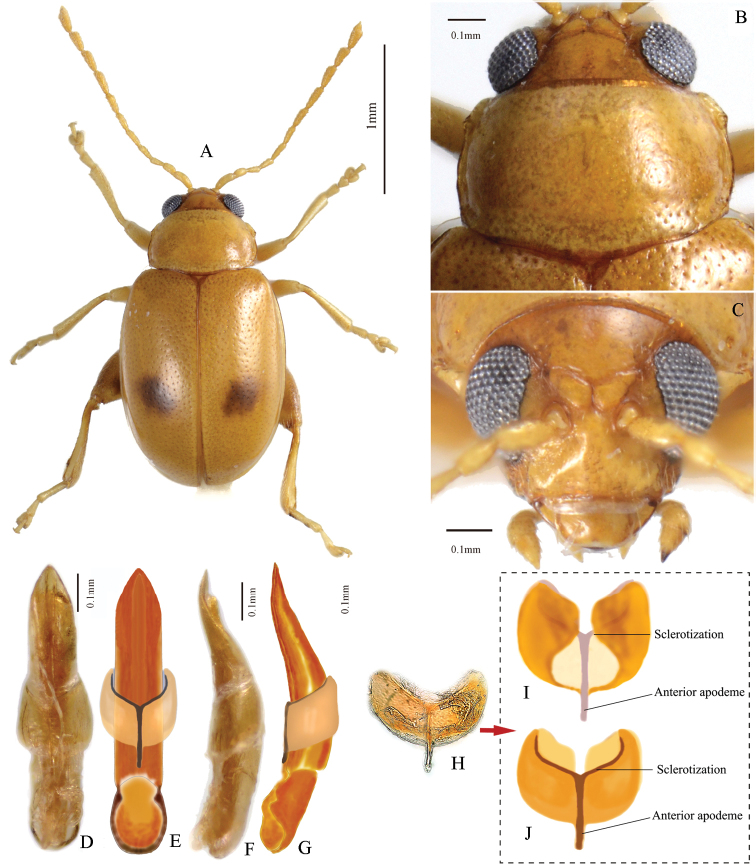
*Lankaphthonabinotata*. Individuals collected in Pingtang island, Fujian Pro., China. **A** Habitus, male **B** prothorax, dorsal view **C** head, frontal view **D** aedeagus, ventral view **E** ventral view of aedeagus, hand drawing, showing sheath-shaped phallobase **F** aedeagus, lateral view **G** aedeagus, hand drawing, lateral view, showing sheath-shaped phallobase **H** phallobase mounted on slide, dorsal view **I** ventral view of phallobase, hand drawing, showing sclerotization **J** dorsal view of phallobase, hand drawing, showing sclerotization.

Morphological study of *Lankaphthonabinotata* unveiled two structures. One of them is previously unknown among leaf beetles: the sheath-shaped phallobase of male genitalia that encircles the aedeagus (Fig. 1H–J), which differs greatly from the usual narrow, mostly twig like phallobase in flea beetles. The phallobase of leaf beetles [also termed ‘tegmen’ (Sharp and Muir 1912; Jolivet et al. 2013), ‘spiculum’, ‘spiculum 1’ or ‘tegminal apodeme’ (Verma 1994, 1996)] is a rather conservative and simple, usually Y-shaped or twig-shaped structure (Sharp and Muir 1912; Chen 1985; Verma 1994, 1996). The ring-shaped phallobase can be found in the genus *Timarcha* (Chrysomelinae) (Sharp and Muir 1912; Jolivet et al. 2013) and rarely in *Chrysolina* (Jolivet et al. 2013). It is considered as a primitive character and usually occurs in basal lineages (e.g., Bruchinae and Donaciinae) of Chrysomelidae and related families (e.g., Cerambycidae) of Chrysomeloidea. In flea beetles, it is usually Y-shaped, and exhibits mild intraspecific variation. In a few flea beetle genera, the shape of the phallobase was used for species identification (Prathapan and Konstantinov 2003; Nadein 2006). However, in *L.binotata*, the phallobase is drastically transformed. Here we describe, discuss and illustrate this unusual structure.

The other structure is a totally unique morphological novelty present only in males – a spoon shaped appendage arising from the first abdominal ventrite (Fig. 3B–E). This unique structure is illustrated and its possible function is discussed.

## Material and methods

The male genitalia were dissected and glued to a paper card pinned beneath the specimens; female genitalia were dissected and mounted on slides in Hoyer’s medium. After photography, they were glued to paper card pinned beneath the specimens. Photos were taken with digital camera Nikon 5200D attached to a Zeiss Axiostar Plus Microscope. The photos of habitus were taken with the 5X objective lens of the same microscope with extra light source softened by semitransparent paper. Hand drawing of habitus of *Lankaphthonabinotata* was painted using water color and Photoshop CS5 (Adobe, San Jose, USA) was used for further rendering. Hand drawings of aedeagus and phallobase of *L.binotata* were entirely composed in Photoshop CS5. The image of *L.binotata* in copula was generated by Autodesk Maya 2014 (Autodesk, Inc., USA) and was edited using Photoshop CS5. Morphological terminology follows Konstantinov (1998). A voucher of the host plant, *Tinosporacordifolia* (Thunb.) Miers (accession number 6488), is deposited in the Calicut University Herbarium, Kerala, India.

Abbreviations of collections:

**BMNH**The Natural History Museum (formerly British Museum), London, UK.

**IZCAS**Institute of Zoology, Chinese Academy of Sciences, Beijing, China.

**KAU** Kerala Agricultural University, Trivandrum, India.

**LMCM** Medvedev collection, Moscow, Russia

**NHMB**Naturhitorisches Museum, Basel, Switzerland.

**TARI**Taiwan Agricultural Research Institute, Taichung, Taiwan, China.

**USNM**National Museum of Natural History, Washington DC, USA.

## Results

### Genus Lankaphthona

Taxon classificationAnimaliaColeopteraChrysomelidae

Medvedev, 2001

Lankaphthona Medvedev, 2001: 162–163. Type species: Lankaphthonamicheli Medvedev, by original designation.
Philotarsa Medvedev, 2009:147. Type species: Philotarsalaosica Medvedev, 2009:147, by original designation. New Synonym.
Longitarsella Medvedev, 2009: 202 (originally as a subgenus of Trachyaphthona). Type species: Philotarsalaosica Medvedev misidentified as Lankaphthonabinotata (Baly, 1876) (originally as Thyamisbinotata Baly, 1876), by original designation. New Synonym.

#### Distribution.

China, Vietnam, Thailand, India, Sri Lanka.

#### Statistics.

8 species.

#### Host plants.

Adults of *L.nigronotatus*, (Jacoby, 1896) feed on the leaves of Menispermaceae. This is the first report of the host plants of the genus.

#### Diagnosis.

The relationships between the flea beetle genera with subparallel ridges on the first abdominal ventrite are in need of comprehensive reevaluation with a rigorous morphological and molecular phylogenetic study. *Lankaphthona* closely resembles *Tegyrius* and *Parategyrius* based on preliminary external morphological characters. These three genera share an overall body shape, long first metatarsomere and similar structure of the head. *Tegyrius* can be separated from *Lankaphthona* by a very narrow orbit (orbit is generally wider in *Lankaphthona*), anteromesal ends of antennal calli acutely narrowed and slightly entering into the interantennal space (in *Lankaphthona* anteromesal ends of antennal calli reach interantennal space, however, does not enter as deep as in the type species of *Tegyrius*, *T.metallicus* Jacoby. This character is difficult to use consistently to separate all known species of *Lankaphthona* and *Tegyrius*); deep sulci delimiting antennal calli (sulci delimiting antennal calli are shallow in *Lankaphthona*); and four labral setae (number of labral setae vary in *Lankaphthona* from 4 to 14). *Lanka* can be distinguished from *Lankaphthona* by the depressed antennal calli separated from each other by the frontal ridge (antennal calli are neither depressed nor separated from each other by the frontal ridge in *Lankaphthona*). In *Philogeus*, antennal calli are separated from each other by the frontal ridge and the bifid claws (in *Lankaphthona*, the antennal calli are not separated completely from each other, and the claws are appendiculate). In *Neorthana*, the frontal ridge is almost rectangular in frontal view and forms an abrupt angle with anterofrontal ridge in lateral view (frontal ridge forms a triangular ridge with anterofrontal ridge and never forms an abrupt angle with anterofrontal ridge in lateral view in *Lankaphthona*). In *Bikasha*, the frontal ridge is narrowed anteriorly above the anterofrontal ridge, the elytral punctures are regularly arranged and the first metatarsomere is shorter (frontal ridge is broadened anteriorly forming a triangular ridge with anterofrontal ridge, elytral punctures confused and the first metatarsomere is longer in *Lankaphthona*). Type material of *Parategyrius* was not available for our study, but specimens identified as congeneric, examined by us, resemble *Lankaphthona* and hence the former is likely to be a senior synonym of *Lankaphthona*.

*Lankaphthona* can be easily separated from *Trachytetra* by the following characters: 1) first metatarsomere almost as long as, or longer than, half of metatibia (first metatarsomere much shorter than half of metatibia in *Trachytetra*); 2) longitudinal subparallel ridges present on first abdominal ventrite (absent in *Trachytetra*); 3) antennal calli moderately developed, indistinctly enter into the interantennal space (antennal calli strongly developed, distinctly enter into the interantennal space in *Trachytetra*). Characters separating *Lankaphthona* and *Lankanella* are a few: frontal and anterofrontal ridges merging into each other forming more or less well-defined Y-shaped structure and confused elytral punctures in *Lankaphthona* and arranged in regular rows in *Lankanella*. In *Lankanella*, frontal and anterofrontal ridges more clearly separated from each other forming more or less well-defined T-shaped structure and the elytral punctures are regularly arranged.

*Lankaphthona* superficially resembles *Aphthona* Chevrolat in having similar characters of head and body shape. However, *Lankaphthona* can be easily differentiated from *Aphthona* by the longer first metatarsomere, presence of antebasal transverse impression on pronotum and the subparallel ridges on first abdominal ventrite. *Lankaphthona* can be confused with *Longitarsus* Latreille due to the general body shape and the elongate first metatarsomere, and there are also several species of *Longitarsus* having black maculation on elytra (e.g. *Longitarsustransversalis* Chen, 1935 from Northern India, *Longitarsusbimaculatus* (Baly, 1874) from China and Japan). However, *Lankaphthona* can be separated from *Longitarsus* by the shape of the frontal ridge and antennal calli, the presence of the antebasal transverse impression on the pronotum and paired intercoxal subparallel ridges on the first abdominal ventrite (both the antebasal transverse impression on the pronotum and the subparallel intercoxal ridges or the appendage on first abdominal ventrite are absent in *Longitarsus*).

Among other Oriental genera, *Doeberlnotus* Prathapan, Konstantinov & Ruan, 2017 and *Sanckia* Duvivier, 1891 have elongate first metatarsomere. *Lankaphthona* can be differentiated from *Doeberlnotus* by the smooth pronotum and body color without metallic luster (pronotum bumpy and body color metallic in *Doeberlnotus*); from *Sanckia* by the glabrous body surface, presence of antebasal groove on pronotum and confused elytral punctation (in *Sanckia*, body surface is covered with dense hair, antebasal transverse impression of pronotum is absent and the elytral punctures are arranged in regular lines.

#### Description.

Small, oblong to oval, convex in lateral view; length 1.5–2.4 mm; 1.6–1.9 times longer than wide, width 0.8–1.3 mm. Color non-metallic, straw brown to red-brown to black.

Head hypognathous. In lateral view, vertex and antennal calli as well as frontal ridge separately form convex lines, meeting point of frontal ridge and antennal calli concave in lateral view. In frontal view, vertex moderately convex, minutely punctate, with obtusely angulate anterior margin. Supraorbital pore situated adjacent to orbital sulcus, with adjacent minute setiferous pores. Antennal callus transverse to oblique, 1–2 times wider than long, separated from vertex. Antennal callus as high as vertex or lower; often a little lower near supracallinal sulcus, much lower near antennal socket than near supracallinal sulcus. Supracallinal sulci distinct, not deep, shallower than orbital sulcus. Anteromesal ends of antennal calli not distinctly angulate, reach up to or slightly entering into interantennal space; antennal calli separated by dorsal end of frontal ridge or narrowly to broadly connected to each other. Supracallinal sulcus transverse to oblique, mostly convex, rarely concave. Midcranial suture absent. Orbit well differentiated from antennal callus by supraorbital sulcus. Subgenal suture well developed along base of mandible. Eye anterolateral, inner margin weakly concave near antennal socket, gently diverging ventrad, vertical diameter 1.2–1.4 times transverse diameter. Distance between eyes 3.0–4.0 times diameter of a socket, 0.9–1.4 times transverse diameter of one eye. Diameter of antennal socket 2.3–4.5 times distance between eye and adjacent socket. Distance between antennal sockets 0.8–1.0 times diameter of a socket. Frontal ridge and anterofrontal ridge together form a triangular ridge, flat anteriorly. Frontoclypeal suture with a row of setae. Antenna filiform, reaching beyond middle of elytra or longer. First and second antennomeres thick, next four or five thin, distal ones slightly thickened. First antennomere longer than second or third separately, little shorter than second and third combined. Labrum with 4–14 setiferous pores arranged irregularly or regularly in transverse row. Maxilla with apical plapomere pointed, shorter or longer than penultimate palpomere. Penultimate palpomere distinctly widened.

Pronotum 1.1–1.6 times wider than long. Antebasal transverse impression sinuate in middle, merge with posterior margin laterally. Almost absent in *Lankaphthonamicheli* Medvedev, however, traces of antebasal transverse impression evident laterally. Width of pronotum anteriorly subequal to width posteriorly. Lateral margin evenly and gently curved. Anterolateral callosity about two times longer than wide, convex and oblique in dorsal view, forms obtuse denticle at pore, pore situated at posterodorsal face of callosity. Posterolateral callosity slightly or strongly protruding, with pore situated laterally. Posterior margin weakly, but distinctly bisinuate, forming lobe in middle. Pronotal punctures minute to small, apparently greater than those on vertex. Anterior coxal cavities open behind. Intercoxal prosternal process extending beyond coxa, apical margin convex, convexly raised along top; apex widened, often with preapical depressions. Shortest width of intercoxal prosternal process more than shortest distance between anterior margin of prosternum and coxal cavity. Prosternum 1.5–1.6 times as long as mesosternum, 0.6 times as long as metasternum. Distance between anterior margin of prosternum to end of prosternal intercoxal process 2.8–6.3 times width of prosternal intercoxal process; width of prosternal intercoxal process 1.2–2.7 times minimum distance between anterior margin of mesosternum to coxal cavity.

Elytra basally wider than pronotum, with basal callus not distinct, without depression posteriorly; humeral callus with or without depression mesally. Elytral punctures confused, fine yet stronger than those on pronotum. Elytral epipleuron extending beyond 3/4 of elytron, hardly reaching apex, subhorizontal to outwardly oblique with maximum width subequal to that of midfemur. Hind wings fully developed. Visible part of mesoscutellum flat, triangular with broadly rounded apex. Mesosternal intercoxal process depressed anteriorly, raised in posterior half. Distance between anterior margin of mesosternum to end of intercoxal mesosternal process 0.8–1.2 times width of mesosternal intercoxal process; width of mesosternal intercoxal process 2.6–4.0 times minimum distance between anterior margin of mesosternum to coxal cavity. Metasternum with anterior margin strongly convexly arched, convexly raised posteriorly, forming paired protuberances raised much above level of metacoxa, as is typical in other genera of the group.

Pro- and mesotibiae dorsally convex, without apical spine. Metafemur robust with anterior margin strongly convex, posterior margin weakly convex. Metatibia in dorsal view distinctly, but weakly curved, with apex directed outwardly. Metatibia almost straight or weakly curved in lateral view; in dorsal view gradually widening from proximal end till it narrows preapically; dorsally convex proximally, turning flat beyond proximal 1/3; distinctly margined both mesally and laterally. Lateral margin with row of pointed bristles, absent proximally up to 2/3 length or less. Mesal margin apically with row of pointed bristles, row of bristles being much shorter than that on lateral margin. Spinules absent on both lateral and mesal margins. Metatibial spine positioned at middle of apex, pointed, shorter than width of metatibial apex. Tarsal articulation on metatibia visible in lateral view, on callosity flanked by flat sclerite on either side. First metatarsomere as long as or longer than half of metatibia, subequal to or longer than next three combined, ventral side densely covered with dense capitate setae. Second metatarsomere apparently longer than third. Third metatarsomere deeply bilobed. Claw appendiculate, apparently shorter than metabitial spine.

Intercoxal part of first abdominal ventrite raised, subparallel ridges on first abdominal ventrite weakly to well developed. Apical tergite of female without longitudinal groove along middle.

Spermatheca with distinct pump, receptacle and duct. Duct not coiled. Receptacle cylindrical, longer than wide, longer than pump.

#### Sexual dimorphism.

In *Lankaphthona*, males can be differentiated from females by the apex of the last abdominal ventrite tri-lobed and incised (evenly convex in female). In *L.nigronotata* (see Fig. 8H) and *L.micheli*, males have much longer antennae than females. A spoon like appendage arising from the first abdominal ventrite in males of *L.binotata* (Fig. 3B–E), is absent in females.

One of the most common characters to separate males and females in flea beetles is the enlarged first protarsomere in males. However, the first protarsomere is not sexually dimorphic in *Lankaphthona*, as males and females have more or less the same sized first protarsomere.

#### Variation.

The maculation on elytra is highly variable in most species of *Lankaphthona*. It could be indistinct or entirely absent in some cases. The color of maculation varies from brown to black in different individuals of the same species. The body color also varies from pale yellow to light brown in some species.

#### Remarks.

*Trachyaphthona* Heikertinger, 1924 was synonymized with *Trachytetra* Sharp, 1886 by Konstantinov and Prathapan (2008: 413). However, Medvedev (2009) treated *Trachyaphthona* as a valid genus name without justification. Here we consider it as a junior synonym of *Trachytetra*.

In most flea beetle genera, the number of labral setae is usually conservative and considered as a more or less reliable generic character. In most cases, there are four (e.g., *Lanka*, *Tegyrius*) or six (e.g., *Chaetocnema* Stephens, *Bikasha*) labral setae. However, the number of labral setae varies greatly in *Lankaphthona*. For instance, there are seven pairs of setae in *Lankaphthonanironotata*, five pairs in *L.micheli* and two pairs in *L.yunnantarsella* sp. nov.

### Key to the species of *Lankaphthona*

**Table:** 

1	Body red brown. Apex of aedeagus in ventral and dorsal views broad, gently narrowed preapically, with emarginate apical margin (Fig. 4F)	***L.micheli* Medvedev**
–	Body straw brown to yellow brown to dark brown, without tinge of red; apex of aedeagus in ventral and dorsal views narrowed and convex or angulate (Figs 1D, 1E, 5G, 6D, 7D)	**2**
2	In lateral view, aedeagus almost straight, only bent ventrally near apex (Figs 6D, 7E)	**3**
–	Aedeagus curved along length in lateral view (Figs 1F, 1G, 5H)	**4**
3	Antennae extremely long, about as long as body; sides of aedeagus parallel in ventral view, apex tri-lobed (Fig. 7F)	***L.yunnantarsella* Ruan, Konstantinov & Prathapan, sp. nov.**
–	Antennae moderately long, about 3/4 the body length; aedeagus with sides slightly sinuate in ventral view (Fig. 6D); apex with acute denticle, not tri-lobed (Fig. 6D)	***L.phuketensis* Gruev, 1989, status restored, comb. nov.**
4	Male with highly specialized spoon-shaped abdominal appendage on first abdominal ventrite (Fig. 3); intercoxal longitudinal ridges on first abdominal ventrite obsolete, almost invisible; eyes not enlarged, distance between eyes to transverse diameter of eye in frontal view ratio: 2.45–2.55	***L.binotata* (Baly, 1876), comb. nov.**
	Male without abdominal appendage on first abdominal ventrite; intercoxal longitudinal ridges on first abdominal ventrite well developed; eyes strongly enlarged, vertex and frons obviously narrowed, distance between eyes to transverse diameter of eye in frontal view ratio: 1.30–1.60	**5**
5	Antennomeres 3 and 4 shorter than antennomere 2 (Fig. 8H); maculation present on posterior part of elytral suture (Fig. 5 D, E)	***L.nigronotata* (Jacoby, 1896), comb. nov.**
–	Antennomeres 3 and 4 longer than antennomere 2; elytral suture without maculation	***L.notatipennis* (Medvedev, 2009), comb. nov.**

Notes: *Lankaphthonacostata* (Medvedev, 2016) comb. nov. (incertae sedis) and *L.cyanipennis* (Medvedev, 2017) comb. nov. (incertae sedis) are not treated here.

### 1. Lankaphthona binotata

Taxon classificationAnimaliaColeopteraChrysomelidae

(Baly, 1876) comb. nov.

Figs 1 , 2 , 3 , 8G

Thyamis binotata Baly, 1876: 583. Type locality: China, Shanghai. Type depository: BMNH. Lectotype designated by Konstantinov and Lingafelter (2002: 214).
Aphthona binotata : Chen 1934: 368.
Zipangia binotata : Konstantinov and Lingafelter 2002: 214.
Trachyaphthona (Longitarsella) binotata : Medvedev 2009: 202.

#### Distribution.

China: Shanghai (Baly 1876), Jiangsu (Ruan and Yang 2015), Fujian (new record).

**Figure 2.**
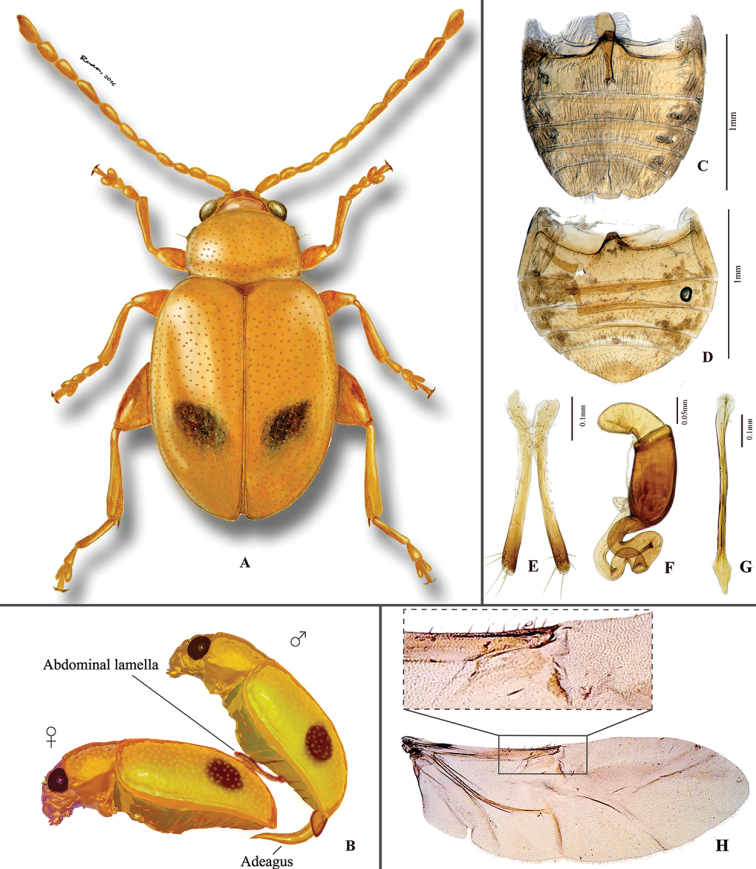
*Lankaphthonabinotata*. **A** Habitus, hand drawing **B** a hypothetical mating diagram: abdominal appendage serving as an auxiliary structure assists the process **C** male abdominal ventrites **D** female abdominal ventrites **E** vaginal palpi **F** spermatheca **G** tignum **H** Hind wing.

**Figure 3.**
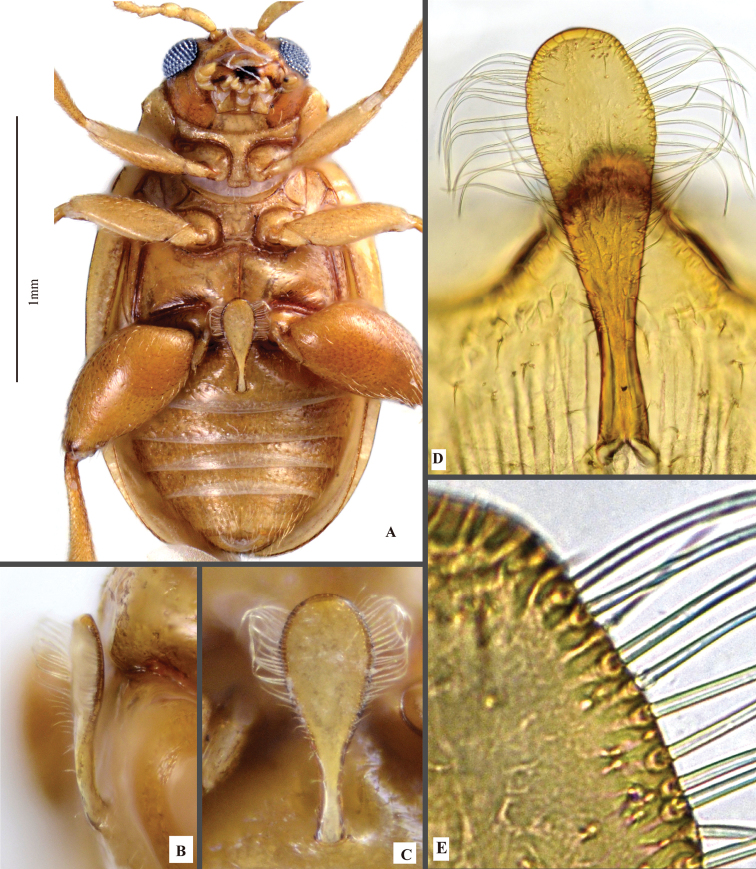
*Lankaphthonabinotata*, showing spoon-shaped abdominal appendage on first abdominal ventrite. **A** Ventral view of male **B** lateral view of abdominal appendage **C** ventral view of abdominal appendage **D, E** close-up view of appendage (mounted on slide and photographed under a light microscope).

#### Description.

Body entirely yellow to yellow brown, each elytron with a black round spot (with indistinct margin) slightly behind middle. Body oval, slightly elongated in dorsal view, dorsum convex in lateral view. Body length: 1.90–2.20 mm. Body width: 1.10–1.20 mm. Body length to width ratio: 1.80–1.85. Pronotum width to length ratio: 1.75–1.80. Pronotum width at base to width at apex ratio: 1.00–1.05. Elytron length (measured along suture) to width of both ratio: 1.25–1.30. Length of elytron to length of pronotum ratio: 3.30–3.40. Width of elytra at base (measured in middle of humeral calli) to width of pronotum at base ratio: 1.10–1.15.

Vertex without punctures, except 2–3 on each side near supraorbital sulcus. Frontal ridge moderately developed, not wide, slightly convex. Sides of frontal ridge without sulci or large punctures, slightly sloping. Antennal calli obliquely elongated, sub-triangular, closely conjoined; lower part narrowed, slightly entering interantennal space. Top of frontal ridge acute, slightly produced between antennal calli. Frontal ridge in lateral view moderately convex. Width of frontal ridge to antennal sockets (counting surrounding ridges) ratio 1.05–1.15. Eyes moderately large, distance between eyes (just above antennal sockets) to transverse diameter of one eye in frontal view ratio: 2.45–2.55. Longitudinal diameter of eye to transverse diameter of eye in frontal view ratio: 2.10–2.15. Distance between antennal sockets to transverse diameter of one antennal socket ratio: 1.05–1.15. Supraorbital and orbital sulci moderately developed. Supraantennal, supracallinal sulci shallow. Frontolateral sulcus obsolete. Orbit wide, as wide as diameter of one antennal socket.

Antennae filiform, moderately long, about 0.8 times body length. Proportions of antennomeres as follows: 12:6:7:8:9:9:10:12:11:10:13. Antennomere 1 almost as long as next two combined. Antennomere 2 robust, slightly shorter than 3 and 4. Length to width of antennomere 9 ratio: 2.30–2.40. Length to width of antennomere 10 ratio: 2.00–2.05. Length to width of antennomere 11 ratio: 2.55–2.60.

Pronotum almost rectangular. Pronotal disc slightly convex. Base of pronotum with a shallow antebasal impression. Pronotal punctures sparse, shallow and minute. Diameter of pronotal punctures 3–4 times smaller than distance between adjacent punctures. Pronotal punctures nearly as large as elytral ones. Anterolateral callosity of pronotum well developed, truncate and elongate, facing anterolaterally. Pronotum parallel sided, not converging forward; lateral margin obviously explanate, slightly sinuate.

Elytron without impressions or ridges. Elytral humeral callus moderately developed. Elytral punctures minute, confused.

Length (not counting trochanter) to maximum width of metafemur ratio: 1.90–1.95. Length to width of metatibia in lateral view ratio: 5.80–5.90. Width of metatibia at base to width at apex in dorsal view ratio: 0.45–0.50. Length of metatibia to length of first metatarsomere ratio: 2.0–2.2. Length of metafemur to metatibia ratio: 1.05–1.10. Length of first metatarsomere to that of second metatarsomere ratio: 2.10–2.20.

Intercoxal ridges on first abdominal ventrite obsolete in both male and female; males with a spoon-shaped appendage arising near hind margin of first abdominal ventrite, produced anteriorly (Fig. 3). Numerous elongate setae present on lateral margin of appendage.

Aedeagus of male robust, oval in cross section. In lateral view, aedeagus robust and sinuate, with apex slightly bent dorsally. Aedeagus, in ventral view, gradually narrowed near apex, apical denticle absent. Ventral groove on aedeagus poorly developed. Phallobase (i.e., tegmen, spiculum) of male genitalia sheath-shaped, encircling middle of aedeagus. Phallobase with a longitudinal sclerotized rod-shaped apodeme in middle, produced anteriorly beyond anterior margin of sheath-like part (Fig. 1I–J: anterior apodeme) and a transverse sclerotization on posterior margin, both together forming a ‘Y’ shaped sclerotization.

Receptacle of spermatheca cylindrical. Spermathecal pump shorter and smaller than receptacle. Basal part of spermathecal duct (between spermathecal gland and receptacle) wide and coiled, longer than receptacle. Apex of spermathecal pump wide, rounded. Lateral margins of vaginal palpus more or less parallel to each other. Vaginal palpus widened near base, weakly sclerotized from base to middle, moderately sclerotized distally. Tignum spear-shaped.

#### Variability.

Depth of pronotal antebasal transverse impression and length of first metatarsomere vary slightly between individuals.

Only a single type of elytal maculation – a round spot with indistinct margin near middle of each elytron – was observed in our study.

#### Type material.

♀ (BMNH), labels: 1) Type H.T.; 2) Baly coll.; 3) *Aphthonabinotata* Baly ♀; 4) A. Warchalowski det. 1965; 5) *Thyamisbinotata* Baly, Shanghai.

#### Material.

4♂4♀ (IZCAS, preserved in ethanol), CHINA, Fujian, Pingtan Island, 5.VI.2014, alt. 200 m, leg. Yongying Ruan; 2♂3♀ (USNM, dry specimens), China, Fujian, Pingtan Isl., WP-449, 25°33.252'N, 119°52.253'E, 5.vi.2014, h = 202 m.

#### Remarks.

*Lankaphthonabinotata* resembles *L.yunnantarsella* Ruan, Konstantinov & Prathapan, sp. nov. due to the similarity in elytral maculation. However, *L.binotata* can be separated from the latter by the much shorter antennae, eyes not prominently enlarged and males with abdominal appendage on first abdominal ventrite.

This species was originally published by Baly (1876) in *Thyamis*. Subsequently, it was placed in *Aphthona* by Chen (1934). Konstantinov and Lingafelter (2002) transferred it to *Zipangia* after they studied the type material. Medvedev (2009) misidentified it and used misidentified specimens as the type for the newly erected subgenusLongitarsella (in genus *Trachytaphthona*). Despite the studies of authors mentioned above, the sheath-shaped phallobase and the highly specialized abdominal appendage on the first abdominal ventrite of males remained unknown prior to this study. We have dissected five males and it turns out that both structures are rather stable in shape.

### 2. Lankaphthona micheli

Taxon classificationAnimaliaColeopteraChrysomelidae

Medvedev, 2001

Fig. 4

Lankaphthona micheli Medvedev, 2001: 163. Type Locality: “Kandy, Sri Lanka”. Type depository: Naturhistorisches Museum, Basel, Switzerland (NHMB).

#### Distribution.

Sri Lanka.

**Figure 4.**
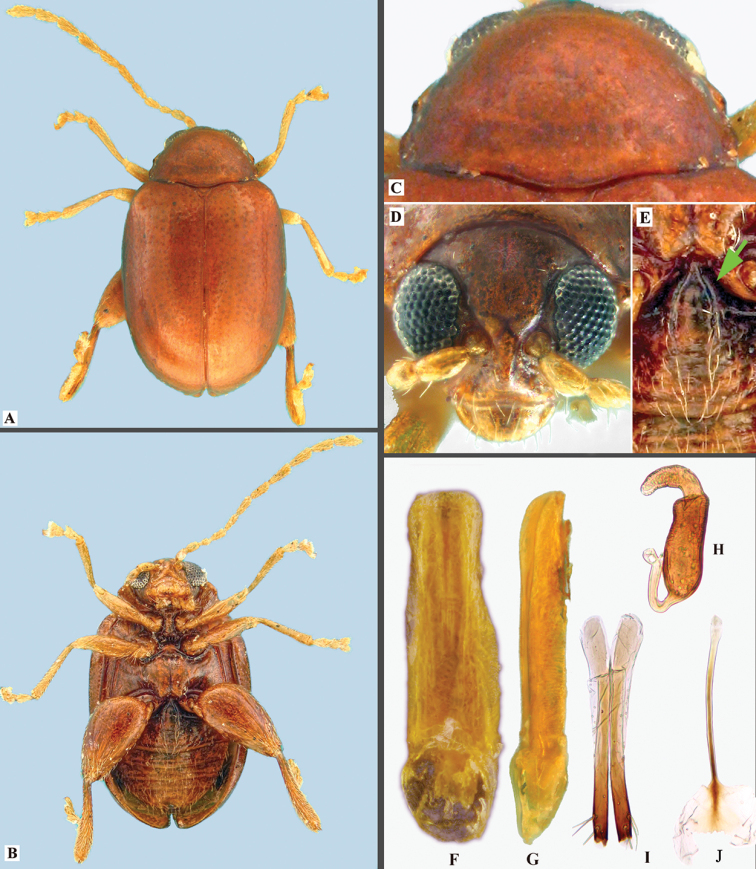
*Lankaphthonamicheli*. **A** Habitus, dorsal view **B** habitus, ventral view **C** pronotum, dorsal view **D** head, frontal view **E** intercoxal ridges on first abdominal ventrite, indicated by arrow **F** aedeagus, ventral view **G** aedeagus, lateral view **H** spermatheca **I** vaginal palpi **J** tignum.

#### Description.

Body rufous brown, except labrum, fore- and middle legs, hind tibia and tarsi lemon yellow. Body broadly oblong in dorsal view, dorsum convex in lateral view. Body apparently larger in female, 1.74–1.84 mm in length, 1.10–1.12 mm in width. Body length to width ratio: 1.55–1.67. Pronotum width to length ratio: 1.40–1.52. Pronotum width at base to width at apex ratio: 1.06–1.13. Elytron length (measured along suture) to width of both ratio: 1.28–1.45. Length of elytron to length of pronotum ratio: 2.96–3.48. Width of elytra at base (measured in middle of humeral calli) to width of pronotum at base ratio: 1.32–1.37.

Vertex with minute and shallow moderate sized punctures. Supraorbital pore adjacent to orbital sulcus, surrounded by shallow groove. Supracallinal sulcus convex, shallow; suprafrontal, supraantennal sulci shallow but more vivid than supracallinal; midfrontal sulcus short, shallow and broader. Antennal callus subtriangular, posteriorly higher than anteriorly, hardly raised above level of vertex, anterior ends slightly enter into interantennal space. Proximal end of frontal ridge narrowed, produced between anterior ends of antennal calli. Frontal ridge well developed, convexly raised between antennal sockets, forms strongly arched convex line in lateral view. Frontal ridge anteriorly flat, forming flat, triangular anterofrontal ridge, raised laterally, flat above clypeus. Antennal socket close to eye. Width of frontal ridge to antennal sockets (counting surrounding ridges), ratio 0.40–0.55. Eyes strongly convex. Distance between eyes (just above antennal sockets) to transverse diameter of eye in frontal view ratio: 0.96–1.80. Longitudinal diameter of eye to transverse diameter of eye in frontal view ratio: 1.39–1.40. Distance between antennal sockets to transverse diameter of one antennal socket ratio: 0.72–0.90. Labrum with about five pairs of irregularly arranged setiferous pores bearing long setae.

Antennae filiform, rather long, about 0.69 times body length in female and 0.82 times body length in male. Proportions of antennomeres as follows: 10: 6.5–8: 4.7–6.7: 7.1–7.3: 8.2–10: 8.7–10: 10–10.6: 10.6–10.7: 10.7–1.18: 10–10.6: 14–15.3. Antennomere 2 robust, 3 shorter and thinner than 2, 4–6 each longer than preceding one. Antennomere 6–9 subequal; 10 shorter than 9. Length to width of antennomere 9 ratio: 2.00–2.22. Length to width of antennomere 10 ratio: 1.64–2.00. Length to width of antennomere 11 ratio: 2.33–2.74.

Pronotum convex, with mixture of shallow, small and minute punctures. Antebasal transverse impression obsolete, leaving an evident trace. Posterior margin weakly bisinuate with lobe in middle. Pronotal punctures distinctly smaller than those on elytra. Anterolateral callosity convex, elongate, with seta bearing pore on upper posterior face. Lateral margin posteriorly narrowed. Posterolateral callosity not protruding.

Elytral punctures irregular, distance between adjacent punctures about 1–3 times diameter of a puncture. Humeral callus well developed, with weak depression posteriorly.

Length (not counting trochanter) to maximum width of metafemur ratio: 1.85–1.94. Length to width of metatibia in lateral view ratio: 5.17–5.60. Width of metatibia at base to width at apex in dorsal view ratio: 0.46–0.60. Length of metafemur to metatibia ratio: 1.72–1.87. Length of first metatarsomere to length of second metatarsomere ratio: 2.57–3.60.

Intercoxal subparallel ridges on first abdominal ventrite well developed (Fig. 4E), barely reaching middle of ventrite. Males without appendage on first abdominal ventrite. Last abdominal ventrite in male with circular depression towards posterior margin, posterior margin with lobe in middle. Last ventrite in female with convex posterior margin as in most flea beetles, apical circular depression and lobe absent.

Aedeagus characteristic: in ventral view, deeply channeled along middle, gently narrowed preapically, apex unusually broad and emarginated in middle; nearly straight in lateral view with apex very slightly curved dorsad. Spermatheca with receptacle about 2.8 times longer than wide, narrowed at distal 1/4, outer margin concave, inner margin convex; pump widened medially, apex rounded without denticle; duct strongly bent towards receptacle, not coiled. Vaginal palpi strongly sclerotized posteriorly than anteriorly; lateral membranous area shorter and narrower than posterior sclerotization. Tignum gently curved; proximal end broadened and lightly sclerotized, distal sclerotization arrow-head shaped.

#### Type material.

Paratype: ♂ (NHMB), labels: 1) Sri Lanka, Kandy, 600 m 1–18.6.1991, N. M. Kolibac leg.; 2) Paratype: *Lankaphthonamicheli* m. L. Medvedev det. 1998.

#### Material.

4 ♂, 1♀ (3 in KAU, 2 to be transferred to USNM) SRI LANKA, Central Prov., 10 km south from Kandy, Uda Peradeniya Vill. Env., 07°15.087'N, 80°37.108'E, 720 m, 24.ii.2013, S. Saluk coll.

#### Remarks.

Medvedev (2001), in his original description of the genus, has stated that the pronotum in *Lankaphthona* is devoid of antebasal transverse impression. However, the pronotum has a very weak antebasal transverse impression that is hard to discern. *Lankaphthonamicheli* can be distinguished from other members of the genus by the red-brown color (all others are yellow brown), feeble antebasal transverse impression on pronotum (well developed in other species) and the unique shape of the aedeagus. The apex of the aedeagus in ventral view is broad and emarginate in *L.micheli*, while the same is narrowed and convex in all other species.

### 3. Lankaphthona nigronotata

Taxon classificationAnimaliaColeopteraChrysomelidae

(Jacoby, 1896) comb. nov.

Figs 5 , 8H

Longitarsus nigronotatus , Jacoby, 1896: 259. Type locality: Myanmar (=Burma), Tharrawaddy. Type depository: BMNH.

#### Distribution.

China: Yunnan (new record); Myanmar (Jacoby 1896), India (Maulik 1926).

#### Host plants.

*Tinosporacordifolia* (Thunb.) Miers (Menispermaceae). Adults feed on the leaves. This is the first report of a host plant of *L.nigronotata*.

#### Description.

Body pale yellow to yellow brown. Each elytron with three brown to black spots: a round one at middle, a longitudinal one on sutural margin near apex and a round one on apex. Legs fulvous, apex of metafemur dorsally brown to black. Body oval, slightly elongate in dorsal view; dorsum convex in lateral view. Body length: 2.00–2.10 mm. Body width: 1.10–1.20 mm. Body length to width ratio: 1.70–1.80. Pronotum width to length ratio: 1.65–1.75. Pronotum width at base to width at apex ratio: 1.10–1.20. Elytron length (measured along suture) to width of both ratio: 1.25–1.35. Length of elytron to length of pronotum ratio: 3.35–3.45. Width of elytra at base (measured in middle of humeral calli) to width of pronotum at base ratio: 1.30–1.40.

Vertex impunctate, except few shallow punctures near eyes. Antennal calli obliquely elongate, sub-triangular, conjoined. Supracallinal, supraantennal and supraorbital sulci well developed, suprafrontal sulcus weak. Frontal ridge proximally acute, produced between antennal calli. Frontal ridge moderately developed, slightly convex. Frontal ridge in lateral view moderately convex. Antennal socket close to eye. Width of frontal ridge to antennal sockets ratio: 0.85–0.90. Eyes strongly enlarged. Distance between eyes (just above antennal sockets) to transverse diameter of eye in frontal view ratio: 1.30–1.40. Longitudinal diameter of eye to transverse diameter of eye in frontal view ratio: 1.65–1.75. Distance between antennal sockets to transverse diameter of one antennal socket ratio: 1.20–1.30. Labrum with about 14 (7 pairs) setiferous pores; 10 long setae arranged in transverse row, additional 4 short ones placed above them.

Antennae filiform, long, about 0.7–0.8 times body length; longer and slender in male. Proportions of antennomeres as follows: 14:7:5:5:11:12:13:14:14:13:15 (measured in male). Antennomere 2 robust, antennomeres 3 and 4 short, subequal, following antennomeres elongate. Length to width of antennomere 9 ratio: 4.90–5.00 (measured in male). Length to width of antennomere 10 ratio: 4.60–4.70 (measured in male). Length to width of antennomere 11 ratio: 4.95–5.05 (measured in male).

Pronotum rectangular, slightly convex; base with distinct, slightly sinuate antebasal impression; punctures sparse, shallow and minute. Diameter of pronotal punctures 3–4 times smaller than distance between adjacent ones. Pronotal punctures nearly as large as elytral ones. Anterolateral callosity of pronotum obliquely truncate. Basal margin slightly convex in middle.

Elytral humeral callus moderately developed. Impressions or ridges absent on elytron. Elytral punctures minute, irregularly arranged.

Length to width of metafemur ratio: 1.95–2.00. Length to width of metatibia in lateral view ratio: 5.65–5.75. Width of metatibia at base to width at apex in dorsal view ratio: 0.40–0.50. Length of metatibia to length of first metatarsomere ratio: 1.80–2.20. Length of metafemur to metatibia ratio: 1.15–1.25.

Subparallel intercoxal longitudinal ridges on first abdominal ventrite hardly reach proximal 1/3 of ventrite.

Aedeagus robust, oval in cross section. Aedeagus in ventral view gently narrowed in middle, apex abruptly narrowed, apical denticle absent; ventral longitudinal groove poorly developed. Aedeagus in lateral view evenly curved with apex bent ventrally.

Receptacle of spermatheca cylindrical, parallel sided. Spermathecal duct wide, strongly curved near middle forming loop towards receptacle. Spermathecal pump shorter and smaller than receptacle, cylindrical, slightly narrowed from base to apex. Vaginal palpus weakly sclerotized anteriorly and medially, strongly sclerotized distally. Vaginal palpus narrowing from base to middle, slightly widening towards apex.

#### Variability.

The elytral spots vary from barely visible, to highly prominent in specimens from Yunnan, China. The aedeagus very slightly varied in shape even in the specimens collected at the same place and time. In the specimens from India, the aedeagus in ventral view with middle part not much narrowed as the specimens from Yunnan.

#### Type Material.

Holotype: ♀ (BMNH), labels: 1) Jacoby coll. 1909-28a; 2) Type H.T.; 3) ♀; 4) *Longitarsusnigronotatus* type Jac.; 5) Tharrawaddy; 6) Examined K. Prathapan 2005.

Paratypes: 4♀ (BMNH), labels: 1) Tharrawaddy; 2) *Longitarsusnigronotatus* type Jac.; 3) Cotype; 4) ♀; 5) Examined K. Prathapan 2005.

#### Material.

CHINA, 13♂1♀ (IZCAS), labels: 1) Yunnan, Xishuangbanna, Menglun, botanical garden, lvshilin, 2009.XI.17, Guo Tang & Zhiyuan Yao leg., 21°54.609'N, 101°17.090'E, 643 m, IZCAS; 2) *Lankaphthonanigronotata* (Jacoby), det. Ruan, 2017; 8♂2♀ (IZCAS), labels: 1) Yunnan, Xishuangbanna, Menglun, 2011.viii, 1088 m; 2) *Lankaphthonanigronotata* (Jacoby), det. Ruan, 2017.

INDIA, Karnataka, 1 ♂ (KAU), labels: 1) Karnataka, Bangalore, 916 m, 17.iv.2000, Prathapan coll., 2) *Lankaphthonanigronotata* (Jacoby), det. Prathapan, 2019; 1 ♂(KAU), labels: 1) Kerala, Chinnar WLS, 10.vi. 2010, Prathapan coll., 2) *Lankaphthonanigronotata* (Jacoby), det. Prathapan, 2019; 2 ♂ (KAU), labels: 1) Kerala, Nelliampathy SeetharGundu, 10°33'21"N, 75°42'53.9"E, 1053 m, 30.x.2010, Prathapan coll., 2) *Lankaphthonanigronotata* (Jacoby), det. Prathapan, 2019; 1♂ (KAU), labels: 1) Vellayani, 8.iv.2010, Prathapan coll., 2) *Lankaphthonanigronotata* (Jacoby), det. Prathapan, 2019; 2 ♀ (KAU), labels: 1) Kerala, Vellayani, 29. vii.2010, Prathapan coll., 2) Host: Tinosporacordifolia, 3) *Lankaphthonanigronotata* (Jacoby), det. Prathapan, 2019; 1 ♀ (KAU), labels: West Bengal, Kalyani, 2–4. xii. 2009, Prathapan coll., 2) *Lankaphthonanigronotata* (Jacoby), det. Prathapan, 2019; 1 ♂, 2 ♀ (KAU), labels: West Bengal, Santhi Niketan, 18.xi.2007, Prathapan coll., 2) *Lankaphthonanigronotata* (Jacoby), det. Prathapan, 2019.

#### Remarks.

*Lankaphthonanigronotata* was originally described in *Longitarsus*. However, this is not a member of *Longitarsus* as evidenced by the prominent antebasal impression on pronotum, explanate lateral pronotal margin, anteromesal ends of antennal calli entering interantennal space and the subparallel intercoxal ridges on the first abdominal ventrite. *Lankaphthonanigronotata* closely resembles *L.notatipennis* Medvedev, 2009, however, it can be separated from the latter by the presence of maculation on posterior part of sutural margin and the apex of the elytra as well as the antennomeres 3 and 4 shorter than antennomere 2.

### 4. Lankaphthona notatipennis

Taxon classificationAnimaliaColeopteraChrysomelidae

(Medvedev, 2009) comb. nov.

Trachyaphthona (Longtitarsella) notatipennis Medvedev, 2009: 202. Type locality: Thailand, Khao Sok. Type depository: Medvedev collection.

#### Distribution.

Thailand.

#### Remarks.

Currently, we do not have access to the type specimens.

We have examined several specimens from Malaysia and India that are very similar to *Longtitarsellanotatipennis*, except for the shape of the aedeagus, which is slightly different from the description provided by Medvedev (2009). The aedeagus is gradually widened from base to rounded apex in the specimens at our disposal, however, according to the original description and illustration provided by Medvedev (2009), the aedeagus is gradually narrowed from base to rounded apex. Hence the types should be examined to ensure identity of the species.

### 5. Lankaphthona phuketensis

Taxon classificationAnimaliaColeopteraChrysomelidae

(Gruev, 1989), status restored comb. nov.

Fig. 6

Longitarsus phuketensis Gruev, 1989: 97. Type locality: Thailand, Phuket Island. Type depository: Gruev Collection, Bulgaria.
Philotarsa laosica Medvedev, 2009:147. Type locality: Laos, Lhammomuang Prov., Ban Khoungham (Nanin). Type depository: L. Medvedev Collection, Russia. New Synonym.
Lankaphthona binotata : Medvedev, 2009: 202 (misidentification)

#### Distribution.

Laos, Thailand.

**Figure 5.**
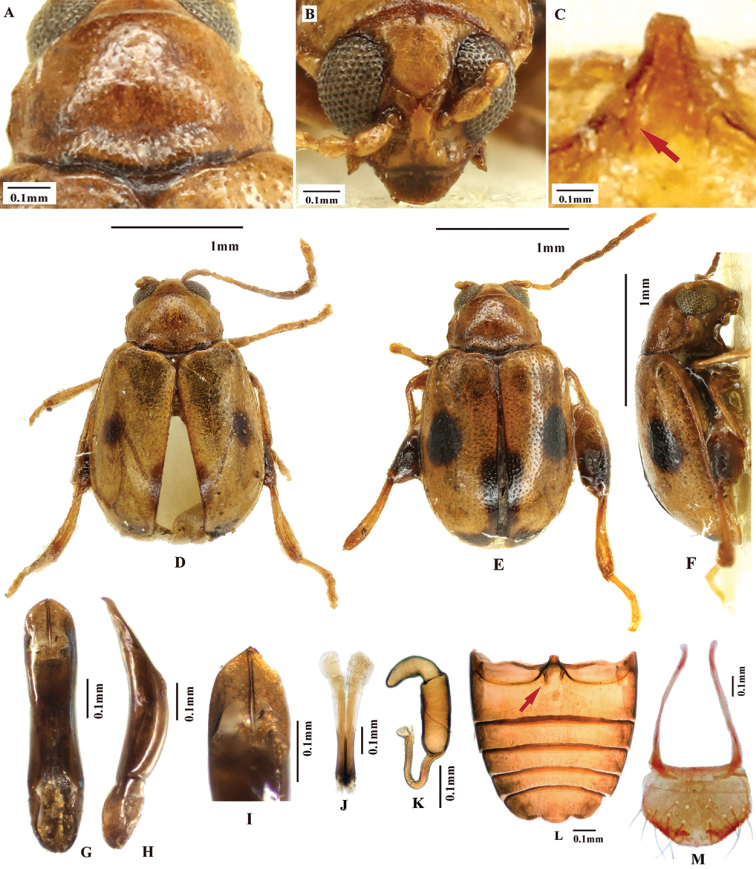
*Lankaphthonanigronotata*. **A** Pronotum, dorsal view, paratype, female **B** head, frontal view, holotype, female **C** intercoxal ridges on first abdominal ventrite, holotype, female **D** holotype habitus, female **E** paratype habitus, female **F** lateral view of paratype **G** aedeagus, ventral view, specimen from Yunnan **H** aedeagus, lateral view, specimen from Yunnan **I** apex of aedeagus, ventral view **J** vaginal palpi **K** spermatheca **L** abdomimal ventrites, male, red arrow indicates intercoxal ridges on first abdominal ventrite **M** labrum, male, showing numerous setae on surface.

#### Description.

Body pale yellow to yellow brown. Each elytron with three brown to black markings: elongate one in middle, a longitudinal one on sutural margin and a round one near elytral humeral calli. Legs fulvous, metafemur dorsally brown to black. Body oval, slightly elongate in dorsal view; dorsum convex in lateral view. Body length: 2.2–2.4 mm. Body length to width ratio: 1.88. Pronotum width to length ratio: 1.66. Pronotum width at base to width at apex ratio: 1.22. Elytron length (measured along suture) to width of both ratio: 1.43. Length of elytron to length of pronotum ratio: 3.53. Width of elytra at base (measured in middle of humeral calli) to width of pronotum at base ratio: 1.27.

Vertex impunctate, except few shallow punctures near eyes. Antennal calli obliquely elongate, sub-triangular, conjoined, entering interantennal space. Supracallinal, supraantennal, suprafrontal and supraorbital sulci well developed. Frontal ridge proximally acute, in lateral view moderately convex, produced between antennal calli. Antennal socket close to eye. Eyes strongly enlarged. Frontal and anterofrontal ridges merge gradually.

Antennae filiform, long, about 0.7–0.8 times body length. Antennomere 2 robust, as long as antennomere 3, slightly shorter than 4, following antennomeres elongate. Length to width of antennomere 9 ratio: 4.11 (measured in female). Length to width of antennomere 10 ratio: 3.77 (measured in female). Length to width of antennomere 11 ratio: 3.81 (measured in female).

Pronotum rectangular, slightly convex; base with distinct, slightly sinuate antebasal impression; punctures sparse, shallow and minute. Diameter of pronotal punctures 3–4 times smaller than distance between adjacent ones. Pronotal punctures smaller than elytral ones. Anterolateral callosity of pronotum obliquely truncate. Basal margin slightly convex in middle.

Elytral humeral callus moderately developed. Elytron without impressions or ridges. Elytral punctures small, irregularly arranged.

Length to width of metatibia in dorsal view ratio: 5.17. Width of metatibia at base to width at apex in dorsal view ratio: 0.39. Length of metatibia to length of first metatarsomere ratio: 1.98.

In ventral view, aedeagus sinuate at sides, dilated before apex, abruptly narrowed near apex, with acute apical denticle. In lateral view, aedeagus straight from base to subapex, with apex bent ventrad.

#### Type material.

Holotype of *Philotarsalaosica* Medvedev, 2009: ♀ (LMCM), labels: 1) Laos, Khammouang Prov., Ban Khounkham (Nahin), 18°13'N, 104°31'E, 200 m, 9.vi.2005, leg. O. Gorbunov.

The type material of *Longitarsusphuketensis* Gruev, 1989 is unavailable to this study. Species concept is based on the author’s (Gruev, 1989) descriptions and illustrations on habitus and aedeagus of holotype.

#### Remarks.

Medvedev (2009: 202) synonymized *Longitarsusphuketensis* Gruev with *Lankaphthonabinotata* (Baly). However, as we mentioned above, in the same work Medvedev (2009) erroneously identified a species that he described as *Philotarsalaosica* as *L.binotata*. Based on the original description and illustration of the habitus and aedeagus of the type provided by Gruev (1989), it is evident that *Longitarsusphuketensis* is clearly different from *Lankaphthonabinotata* and can be separated by the following characters: apical and basal spots present on elytron (apical and basal spots on elytron are absent in *Lankaphthonabinotata*); aedeagus straight from base to subapex in lateral view, with acute apical denticle in ventral view (in *Lankaphthonabinotata*, aedeagus curved ventrally from base to near apex, without acute apical denticle); body length slightly larger (2.40 mm in *Longitarsusphuketensis*, 1.90–2.20 mm in *Lankaphthonabinotata*).

Based on Medvedev’s suggestion of the synonymy of *Longitarsusphuketensis* and *Philotarsalaosica* (erroneously identified as *L.binotata*) and our observations of the holotype of *Philotarsalaosica*, we here synonymize *Philotarsalaosica* Medvedev, 2009 with *Longitarsusphuketensis* Gruev, 1989.

*Lankaphthonaphuketensis* is close to *L.notatipennis* in the similar pattern of maculation on elytron (e.g., having apical and basal spots), the type localities of the two species are also very close to each other. But *L.phuketensis* can be differentiated from *L.notatipennis* by the aedeagus straight from base to near apex in lateral view, with acute apical denticle in ventral view (in *L.notatipennis*, aedeagus curved ventrally from base to near apex, without acute apical denticle in ventral view).

### 6. Lankaphthona yunnantarsella

Taxon classificationAnimaliaColeopteraChrysomelidae

Ruan, Konstantinov & Prathapan sp. nov.

http://zoobank.org/22ABFD57-B137-42B1-A5D9-646535A5D0B8

Figs 7 , 8A–F

#### Type Locality.

China: Yunnan (Xishuangbanna).

#### Etymology.

The name is derived from the type locality and the elongate first metatarsomere of this species.

#### Distribution.

China: Yunnan.

#### Diagnosis.

*Lankaphthonayunnantarsella* Ruan, Konstantinov & Prathapan, sp. nov. is close to *L.binotata* (Baly) due to the similarity in body color and elytral maculation. However, it can be separated from *L.binotata* (Baly) by the strongly enlarged eyes, narrow vertex and frons, longer antennae and the absence of abdominal appendage on first abdominal ventrite in male.

**Figure 6.**
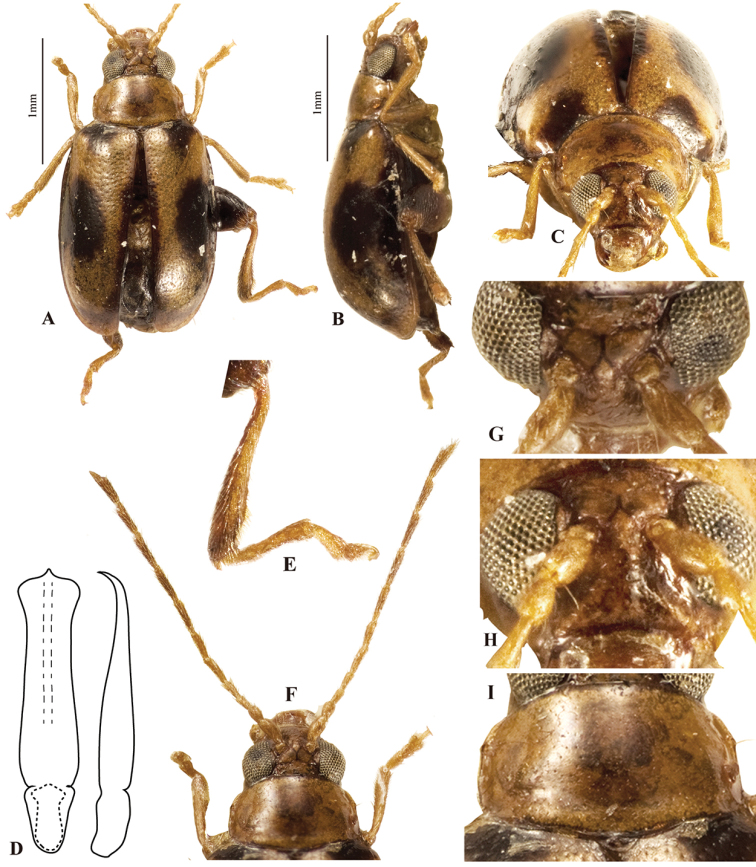
*Lankaphthonaphuketensis* (Gruev, 1989) (= *Philotarsalaosica* Medvedev, 2009). **A–C** and **E–I** are photo of holotype of *Philotarsalaosica*, female. **A** Habitus, dorsal view **B** habitus, lateral view **C** habitus, frontal view **D** shape of aedeagus, hand drawing, based on illustration provided by Gruev (1989)**E** metatibia and metatarsomere, dorsal view **F** head, antennae and pronotum, dorsal view, Holotype **G** head, showing supraantennal calli, dorsal view **H** head, frontal view **I** pronotum, dorsal view.

**Figure 7.**
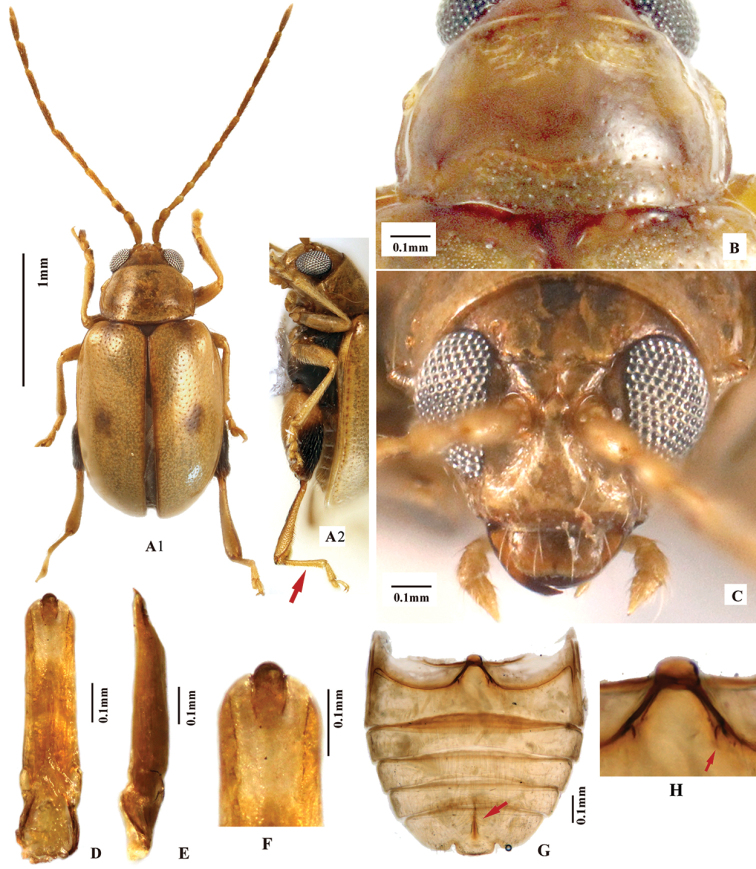
*Lankaphthonayunnantarsella* sp. nov. **A** Holotype habitus, male, dorsal view (**A_1_**) and lateral view (**A_2_**) **B** paratype, prothorax, dorsal view **C** paratype, head, frontal view **D** paratype, aedeagus, ventral view **E** paratype, aedeagus, lateral view **F** paratype, apex of aedeagus, ventral view **G** paratype, abdominal ventrites of male, ventral view **H** paratype, showing longitudinal intercoxal ridges on first abdominal ventrite of male.

#### Description.

Body pale yellow to yellow; meso- and metasternum and apex of metafemur dark brown dorsally; two brown to black spots with indistinct margin present on each elytron: one situated at middle and other at base on mesal side of humerus. Body oval, slightly elongate in dorsal view, dorsum convex in lateral view. Body larger in female, 1.70–2.10 mm in length, 0.80–1.10 mm in width. Body length to width ratio: 1.85–1.95. Pronotum width to length ratio: 1.65–1.75. Pronotum width at base to width at apex ratio: 1.05–1.10. Elytron length (measured along suture) to width of both ratio: 1.30–1.35. Length of elytron to length of pronotum ratio: 3.15–3.25. Width of elytra at base (measured in middle of humeral calli) to width of pronotum at base ratio: 1.30–1.40.

Vertex without punctures, except 2–3 on each side near supraorbital sulcus. Frontal ridge moderately developed, evenly convex. Sides of frontal ridge without sulci or punctures, oblique. Antennal calli obliquely elongate, sub-triangular, conjoined, well delimited with supracallinal, midfrontal, supraantennal, and suprafrontal sulci. Top of frontal ridge acute, produced between antennal calli. Frontal ridge in lateral view moderately convex. Antennal socket close to eye. Width of frontal ridge to antennal sockets (counting surrounding ridges) ratio 0.50–0.60. Eyes strongly enlarged. Distance between eyes (just above antennal sockets) to transverse diameter of eye in frontal view ratio: 1.50–1.60. Longitudinal diameter of eye to transverse diameter of eye in frontal view ratio: 2.00–2.10. Distance between antennal sockets to transverse diameter of one antennal socket ratio: 0.85–0.95. Labrum with four setiferous pores bearing long setae.

Antennae filiform, rather long, about 0.9 times body length in female and 1.0 times body length in male. Proportions of antennomeres as follows: 13:7:8:9:12:11:15:15:15:14:19. Antennomere 2 robust, shorter than antennomere 3 and 4, following antennomeres slender. Length to width of antennomere 9 ratio: 5.85–5.90. Length to width of antennomere 10 ratio: 3.65–3.70. Length to width of antennomere 11 ratio: 5.50–5.55.

Pronotum more or less rectangular, slightly convex, antebasal transverse impression poorly to well developed. Pronotal punctures shallow and minute, slightly larger near antebasal transverse impression. Diameter of pronotal punctures 3–4 times smaller than distance between them. Pronotal punctures nearly as large as elytral ones. Anterolateral callosity of pronotum well developed, truncate and elongate, facing anterolaterally. Lateral margins of pronotum slightly sinuate, not converging anteriorly, with lateral margin obviously explanate. Pronotal base slightly convex at middle.

Elytral humeral callus moderately developed. Impressions or ridges absent on elytron. Elytral punctures minute, confusedly arranged. Elytra at base wider than pronotum.

Length (not counting trochanter) to maximum width of metafemur ratio: 1.95–2.00. Length to width of metatibia in lateral view ratio: 5.65–5.75. Width of metatibia at base to width at apex in dorsal view ratio: 0.45–0.50. Length of metatibia to length of first metatarsomere ratio: 1.80–1.90. Length of metafemur to metatibia ratio: 1.15–1.25. Length of first metatarsomere to length of second metatarsomere ratio: 2.50–2.55.

Subparallel intercoxal ridges on first abdominal ventrite short, not reaching proximal 1/3 of ventrite. Males without appendage on first abdominal ventrite.

Aedeagus nearly parallel sided in ventral view, with basal part slightly wider; apex tri-lobed, with sulci between middle and lateral lobes (Fig. 7F). Ventral groove present. In lateral view, aedeagus almost straight with base and apex very slightly curved ventrally.

#### Variability.

**Figure 8.**
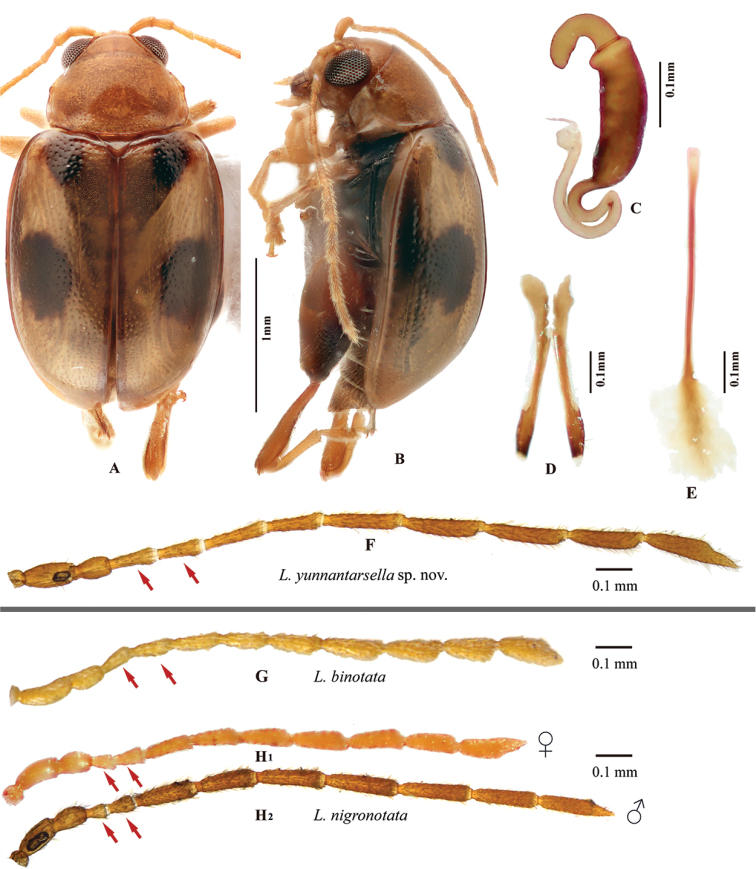
**A–F***Lankaphthonayunnantarsella* Ruan, Konstantinov & Prathapan, sp. nov. **G***L.binotata* (Baly) **H_1_–H_2_***L.nigronotata* (Jacoby). **A** Paratype habitus, female, dorsal view **B** paratype habitus, female, lateral view **C** paratype, spermatheca **D** paratype, vaginal palpi **E** paratype, tignum **F** paratype, male antenna **G** male antenna **H** antenna, female (**H_1_**) and male (**H_2_**).

The number and position of elytral spots are consistent in all the specimens studied: one situated near middle and the other at the base on mesal side of humerus. However, spots varied from brown to black and poorly visible (e.g., in holotype, Fig. 7A) to distinctly prominent (e.g., in paratype, female, Fig. 8A, B). The basal spot on the mesal side of the humerus is almost invisible in the holotype, but for a trace of brown maculation. In female, an additional longitudinal spot present near lateral margin of elytra, merging with the middle spot.

#### Type material.

Holotype: ♂ (IZCAS), labels: 1) Yunnan, Xishuangbanna, Menglun, botanical garden, lvshilin, 2009.xi.17, Guo Tang & Zhiyuan Yao leg., 21°54.609'N, 101°17.090'E, 643 m, IZCAS; 2) Holotype, *Lankaphthonayunnantarsella* sp. nov. Des. Ruan, Konstantinov & Prathapan, 2018.

Paratypes: 2♂ (1 in TARI, 1 to be transferred to USNM), labels: 1) Yunnan, Xishuangbanna, Menglun, botanical garden, lvshilin, 2009.xi.17, Guo Tang & Zhiyuan Yao leg., 21°54.609'N, 101°17.090'E, 643 m, IZCAS; 2) Paratype, *Lankaphthonayunnantarsella* sp. nov. Des. Ruan, Konstantinov & Prathapan, 2018; 1♂1♀ (IZCAS), labels: 1) Yunnan, Xishuangbanna, Menglun, 2011.viii, 1088 m; 2) Paratype, *Lankaphthonayunnantarsella* sp. nov. Des. Ruan, Konstantinov & Prathapan, 2018.

#### Remarks.

The specimens of *L.yunnantarsella* sp. nov. Ruan, Konstantinov & Prathapan and *L.nigronotatus* were collected from the same location in Yunnan province, China. Hence it is very easy to confuse these two species due to their similarity in body shape, color and elytral maculation. However, they can be carefully separated based on external characters. In *L.yunnantarsella* antennae are about 0.9–1.0 times body length, antennomere 2 shorter than antennomere 3 and 4 and each elytron with two dark spots (in males), one situated at middle and the other basally on mesal side of humerus. In *L.nigronotatus*, antennae are about 0.8 times body length, antennomere 2 longer than antennomere 3 and 4; and elytron with three dark spots: one situated at middle, one elongate spot on sutural margin near apex and the third one at apex.

## Discussion

### Sheath-shaped phallobase of *L.binotata* (Fig. 1D–J)

The phallobase exhibit very limited variation within or between genera in flea beetles. However, it offers distinct but highly stable variation between family groups of Chrysomeloidea and Cerambycoidea. Hence it is extensively used in phylogenetic studies on higher classification of leaf beetles and allied families (e.g. Chen 1985; Verma 1996). Chen (1985) identified four types of male genitalia in Chrysomeloidea (*sensu lato*): cerambycid, megalopodid, sagrid, chrysomelid and eumolpid.

According to Crowson (2013), the sheath-like phallobase has evolved polyphyletically in many families of Coleoptera (e.g., Histeridae, Buprestidae, Bostrychidae and Trogossitidae). Within Chrysomeloidea, gutter-shaped fused lateral lobes encircling median lobe have been reported in the bruchid genus *Caryopemon* Jekel (Li et al. 2016). However, in Chrysomelidae, the phallobase is long, slender and T- or Y- shaped (Verma et al. 1996) and is the basic structure in most subfamilies (e.g., Chrysomelinae, Galerucinae, Synetinae, Cassidinae). The structure of the phallobase in *L.binotata* is more primitive and closely resembles a modified abdominal sternite rather than a regular T- or Y-shaped chrysomelid phallobase. The sheath-like phallobase of *T.binotata*, which apparently is a homoplasy, indicates that the phallobase can be modified and highly specialized even in highly evolved species or genera.

The chrysomelid phallobase plays a vital role in the reversal of the aedeagus as both protractor and retractor muscles of aedeagus are attached to it (Verma 1994, 1996). Apparently, the sheath-like phallobase (spiculum) too performs the same function.

The shape of the phallobase in *L.binotata* also indicates that it is a modified true abdominal sternite or tergite. As the structure has an anteriorly produced sclerotized rod-shaped apodeme in middle (Fig. 1I–J: anterior apodeme), it supports the theory that the phallobase of Coleoptera has originated from sternite 9 with a ‘median anterior apodeme’ (Crowson 2013).

### Abdominal appendage of *L.binotata* (Fig. 3)

Several secondary sexual characters are known in flea beetles. The most common ones are the following: the larger body size in female; sexually dimorphic shape of posterior margin of last visible abdominal ventrite and pygidium; and first protarsomere much more enlarged in males than in females in many genera (Konstantinov and Lingafelter 2002). ‘Abdominal appendages’ are present in specific groups of chrysomelids, especially Galerucini. For instance, in males of *Pseudoluperustuberculatus* (Blake) (Galaerucini), two short and simple lamellae are present on the second abdominal ventrite; males of *Scelida* Chapuis also has odd ventral appendages (Riley et al. 2002); in *Hoplasoma* Jacoby, some species tend to have 1 or 2 pairs of processes on abdominal ventrite (Bezděk and Zhang 2007; Bezděk 2014). Mohamedsaid and Furth (2011) reported that there are 66 species from 18 different genera of Galerucini having lamella-shaped abdominal ornaments.

Such specialized abdominal appendages are rare in flea beetles. There are two Australian flea beetle (Alticini) genera *Axillofebra* Samuelson and *Profebra* Samuelson with extra structures on procoxa, which are produced over part of the trochanterofemoral articulation (Samuelson 1969), but these structures are simple and do not seem to be specialized.

Morphological structures, similar to that in the male of *L.binotata*, occur in *Haplosomoides* Duvivier (Galerucini). In some species of *Haplosomoides*, the structure is prominent and delicate, and is used as one of the main diagnostic characters to delimit species and species groups (e.g., Lee et al. 2011). Although origin of the abdominal appendages in *Haplosomoides* and *L.binotata* are probably polyphyletic, they are almost morphologically indistinguishable: both are highly specialized, spoon-shaped, with elongate setae on apical part.

The evolutionary significance or function of the spoon-shaped abdominal appendage still remain unexplained. The true function of the structure should be tested empirically. However, as it is currently only known in males, we presume that it is related to copulation, most probably as an auxiliary structure in the mating process (shown in Fig. 2B).

## Supplementary Material

XML Treatment for Lankaphthona

XML Treatment for Lankaphthona binotata

XML Treatment for Lankaphthona micheli

XML Treatment for Lankaphthona nigronotata

XML Treatment for Lankaphthona notatipennis

XML Treatment for Lankaphthona phuketensis

XML Treatment for Lankaphthona yunnantarsella
